# How Managers Perceive and (Do Not) Participate in Health Promotion Measures—Results from a Cross-Sectional Mixed-Methods Survey in a Large ICT Company

**DOI:** 10.3390/ijerph18189708

**Published:** 2021-09-15

**Authors:** Kristina Schubin, Holger Pfaff, Sabrina Zeike

**Affiliations:** Institute of Medical Sociology, Health Services Research and Rehabilitation Science, Faculty of Human Sciences, Faculty of Medicine and University Hospital Cologne, University of Cologne, 50933 Köln, Germany; holger.pfaff@uk-koeln.de (H.P.); sabrina.zeike@vivalue-gmbh.de (S.Z.)

**Keywords:** manager, health promotion, participation, company, work, occupational health

## Abstract

Managers often face stress and high work demands. Yet they have received limited attention as targets of workplace health promotion measures (HPMs). This study’s primary objective (1) is to examine managers’ self-reported participation in HPMs and factors associated with HPM participation. The secondary objective (2) is to examine managers’ perceptions of their working conditions. A cross-sectional mixed-methods online survey was conducted with a nonrandom sample of 179 managers in a large German ICT company. Stepwise logistic regression and qualitative content analysis were used for data analysis. Quantitative findings revealed that 57.9% of managers had not participated in HPMs yet. “Workload relief through digital tools” resulted as a significant predictor of managers’ previous HPM participation (OR: 2.84, 95% CI: 1.42–5.66). In qualitative findings, workload, time, lack of knowledge, and lack of demand were reported as participation barriers (1). Managers reported that work facility traits, workload, social support, and corporate culture should be improved to make their working conditions more health-promoting (2). These findings suggest that providing adequate organizational working conditions may help improve managers’ HPM participation rates and their perception of health-promoting work.

## 1. Introduction

Despite researchers’ agreement that managers should be role models regarding health-promoting behavior [1], we know little about managers’ participation in workplace health promotion measures (HPMs). Since the working world is changing rapidly [2,3,4], there is a continuous need to assess factors influencing availability of and participation in HPMs across occupational groups [5]. Managers in particular face high workload [6], (techno-)stress [4,7,8,9], and the challenge of leading digital transition in organizations [10,11,12], making them special targets for HPMs. Workplace health promotion is defined by all joint measures of employers, employees, and society aimed at improving health and wellbeing at the workplace [13,14]. While the participation rate in HPMs is a key indicator for their effectiveness, it typically amounts to only 20–40% of staff participating [15,16]. Some previous findings suggest that managers are more likely to participate in HPMs or report HPM availability more often compared to nonmanagerial employees [17,18,19]. Still, how managers perceive implementation of workplace health promotion [20,21,22,23] or what factors influence employees’ HPM participation from managers’ perspectives [24,25,26,27,28] has been investigated far more thoroughly than managers’ own HPM participation. Hence, this study aims to add to this state of research.

Existing multilevel theories about workplace health promotion and use of health services suggest that factors on the individual, interpersonal, and organizational level influence HPM participation [29,30,31]. We employ Andersen’s model of health service utilization to guide our statistical regression analysis and discuss findings [31]. Andersen’s model explains health care utilization using individual and contextual characteristics that predispose or enable people with health needs to seek health care measures. Predisposing characteristics, enabling resources, impeding factors, and perceived health can be used as categories to examine individual and organizational level factors associated with managers’ HPM participation. Although we do not intend to validate or disprove Andersen’s model, its multilevel structure helps put findings into perspective and extends previous research.

The current state of research demonstrates that contexts, measures, and target groups of HPM participation studies are highly heterogeneous. On the individual level, there are mixed findings whether employees of older age, female gender, higher educational level, and a good health status are generally more likely to participate in HPMs [15,16,18,32,33,34,35]. However, there is consistent evidence that health-oriented awareness and behavior [16,36], work demand satisfaction [32], knowledge about availability of HPMs, motivation, self-efficacy, and expected outcomes [25,36,37,38] affect participation positively. On the organizational level, factors such as the company’s physical environment [33,39], social and cultural environment [35,37,39,40], working structures [18,24,37], the HPM design [15,36,41], fit to employees’ needs and preferences [34], and financial incentives [39,42] influence participation. Consequently, both individual and organizational factors should be considered in the study of managers’ HPM participation. As there is lacking understanding how organizational-level characteristics predict participation in HPMs and effectiveness of HPMs [36,39], organizational factors such as managers’ working conditions should be studied more intensely.

Thus, the primary objective (1) of this study is to examine managers’ self-reported participation in HPMs and factors associated with their HPM participation. The secondary objective (2) is to examine managers’ perceptions of their working conditions. For this, we employ a mixed-methods exploratory survey [43]. Using a mixed-methods survey aims at enhancing both quantitative and qualitative findings. The purpose is to achieve complementarity and expansion of findings to extend the answer to the research objectives. Consequently, quantitative and qualitative findings should address components of both objective (1) and (2). While the study is quantitatively driven, the authors assign an equal status to quantitative and qualitative findings (QUAN + QUAL [44,45]). Compared to existing research, this mixed-methods study adds value by focusing on managers as a particular target group of workplace health promotion.

## 2. Materials and Methods

### 2.1. Study Context

This article is part of a larger study with two components; we focus on the second. The first study component was the outcome evaluation of a mindfulness training program for managers. The training was pilot-tested in a large German ICT company from October to December 2019. The evaluation aimed at assessing training effects on participant outcomes such as health status or mindfulness. However, we do not investigate this component in the present article. Instead, we focus on the second component: the analysis of a nonparticipation survey that was conducted in the same ICT company from November 2019 to January 2020. The purpose of the survey was to provide guidance for the company’s health managers to improve the occupational health management and ICT managers’ overall participation in HPMs. Managers who did not participate in the mindfulness training represented this survey’s target group. Consequently, the data basis for this article consists of a restricted subsample of all managers who did not participate in the specified training. However, independent of the training, this article aims to examine these managers’ general participation in HPMs (objective 1) and their health and work situation (objective 2). Thus, this article does not address nonparticipation in the specified training in particular but employs an overarching perspective on managers’ general HPM participation and working conditions. The ICT company offers a wide range of HPMs such as workout in gyms, running events, or measures aiming at mindfulness and resilience. Approval for this study was granted by the Ethics Committee of the Medical Faculty of the University of Cologne (project identification code: 19-1476).

### 2.2. Survey Development

The authors developed and pilot-tested a survey cooperating with three upper-level health managers in the ICT company. The survey assessed managers’ perceptions of factors for (non)participation in HPMs, preferences for HPMs, and perceptions of their health and working conditions. A mixed-methods survey approach was chosen to obtain distributions of managers’ characteristics, while at the same time providing managers with the opportunity to share their experiences in more depth. The survey combined Likert scales, closed questions with categorial response options, and open-ended questions. The survey did not include obligatory questions. During pilot-testing, one manager offered a slight adaptation of the wording and online layout of the survey via email. In a meeting with the authors, another manager made more explicit suggestions for questions regarding perceived working conditions and factors of (non)participation in HPMs. Overall, the pilot-testing managers considered the survey short and comprehensible. The final survey comprised 29 questions in total. Out of these, five questions were open-ended and 14 were conditional. Out of 24 closed questions, six questions offered an additional open-response item. Five questions each aimed at demographical and occupational information, health-related characteristics, and work-related characteristics. Data on HPM participation were collected with a total of 13 questions.

### 2.3. Sample and Data Collection

In this study, 1705 upper-level managers from a German ICT company served as the study population. Upper-level managers were defined as executives with high responsibilities leading lower-level managers. These managers were invited to participate in the survey with a company internal one-time email sent by the Human Resources Department. The email contained a link to the online survey which was administered using the web-based tool LimeSurvey (LimeSurvey GmbH, Hamburg, Germany). Data were collected from November 2019 to January 2020. Out of 239 managers accessing the survey, 179 completed the full 7-page questionnaire. Managers who did not complete the survey were excluded from the analysis sample (*n* = 60). Out of all excluded cases, 46 managers quit the survey immediately after accessing the landing page. The remaining 14 cases quit the survey on the following pages. Thus, 179 participants served as the analysis sample (response rate = 10.5%). The participants agreed to analysis and anonymous publication of collected data for research purposes. The data sets were not passed on to the company.

### 2.4. Measures

#### 2.4.1. Sociodemographic Characteristics, Health- and Work-Related Scales

The collected sociodemographic data comprised age groups (<18 years, 18–24 years, 25–44 years, 45–64 years, and >65 years), gender (female and male), and management level (top, middle, and low) as category variables. Managerial experience (in years) was included as a continuous variable. For category variables, participants had the opportunity to choose “not specified” as a response.

Health- and work-related characteristics were measured by means of self-rated scales. Data were collected on the current subjective wellbeing, overall health status, and work intensity. Subjective wellbeing was measured using the German version of the World Health Organization Well-Being Index (WHO-5) [46]. The WHO-5 is a validated and widely used self-observation measure assessing psychological wellbeing within the last two weeks. The WHO-5 comprises five positively worded items on a six-point Likert scale, e.g., about one’s mood or vitality (0 = “not present” to 5 = “constantly present”). Based on established WHO-5 cutoff scores indicating poor or high psychological wellbeing [47,48], we dichotomized subjective wellbeing to distinguish managers with poor wellbeing (WHO-5-score ≤ 50) from those with high wellbeing (WHO-5-score > 50). The scale’s internal consistency was Cronbach’s α = 0.89.

The overall self-rated health status was measured by a five-point Likert scale with one item, based on the German Socio-Economic Panel (SOEP) [49]. Participants were asked to rate their current health status: “In general, how would you describe your current health status?’’ (1 = “bad” to 5 = “very good”).

Lastly, a scale was used to measure work intensity. The scale was based on the Compendium of Valid Employee Key Performance Indicators (MIKE) [50]. The scale aims to evaluate the relationship between working situation and a person’s health and thus identify a possible misfit of decision latitude and work intensity [51]. The scale consists of six items, e.g., “I am frequently under time pressure at work” or “I often have to complete many tasks simultaneously”. Participants rated their level of agreement with each item on a four-point scale (1 = “strongly disagree” to 4 = “strongly agree”). In this study, we included an additional item (“My job is very mentally demanding”) to account for the psychological aspect of mangers’ work intensity in the ICT industry. The internal consistency of the resulting seven-item scale was Cronbach’s α = 0.79.

#### 2.4.2. HPM Participation and Working Conditions

A mixture of closed questions with dichotomous response options and open-ended questions aimed at exploring managers’ HPM participation and perceptions of their working conditions. Managers were asked “Do your working conditions promote healthy working?” (yes, no). Regardless of the answer, managers were then presented with an open-ended question: “From your perspective, which improvements are necessary to make your working conditions more health-promoting?”. Managers were also asked “Do digital tools help you in relieving your daily workload?” (yes, no), to account for the impact of ICT demands in managers’ work. All managers were asked “Have you participated in HPMs before?” (yes, no) to assess previous HPM participation. If managers reported they had participated in HPMs before, they were presented with the opportunity to name up to three measures they had previously attended (“Which workplace HPMs have you attended?”).

The remaining questions in the survey were conditional. Managers were presented with these questions depending on their response in a previous question. If managers specified they did not know about the initial mindfulness-based training that was currently offered in the company, managers were then asked “Do you wish to participate in HPMs more often?” (yes, no, don’t know). If managers did wish to participate more in HPMs, they were asked “What keeps you from doing so currently?” (open-ended question). This was followed by the question “Which HMPs would interest you?” (category multiple response option). Managers could then choose multiple responses from “Face-to-Face workshops”, “Digital measures”, “Individual coaching”, “Formal exchange with colleagues”, “Blended learning”, and “Other” (with an additional open field to specify other HPM modes). If managers specified that digital tools help relieve their daily workload, they could then name specific digital tools in the subsequent open-ended question “Which digital tools help relieve your daily work?”

### 2.5. Data Analysis

The analysis of quantitative and qualitative data was conducted independently. Analysis of qualitative data followed analysis of quantitative data. The findings are integrated in the discussion.

#### 2.5.1. Quantitative Analysis

Descriptive statistics were used to report sample characteristics. Stepwise multivariate logistic regression models were used to analyze odds ratios for managers’ previous HMP participation. The sequence of added variables was based on Andersen’s model of health service utilization [31]. In this study, we defined health service utilization as previous participation in HPMs. Andersen’s model suggests a sequence in which variables on the individual and contextual level affect health service utilization. Variables are allocated to predisposing characteristics, enabling resources or need factors, and then included in a subsequent modelbuilding process. This approach enables identifying when an effect is explained by another effect in predicting previous participation in HPMs. In addition to an enabling resource, we added an impeding factor to the model.

The dependent variable in the regression was defined by the dichotomous response to the question “Have you participated in HPMs before?” (yes, no). Three regression models were progressively adjusted. Model 1 comprised predisposing individual characteristics (age group, gender, and management level). For the regression analysis, age was dichotomized into two groups (25–44 years and 45–64 years). Further, the management level was dichotomized (low and high) by summing the middle and lower management level to provide sufficient sample size per category. Model 2 added subjective wellbeing. Due to its high validity and established cutoff scores [48], the dichotomous variable wellbeing (WHO-5-score low vs. high) was chosen as a predictor indicating health services need. Finally, Model 3 added one enabling resource (workload relief through digital tools) and one impeding factor (work intensity). Workload relief through digital tools (“Do digital tools help you in relieving your daily workload?”; yes, no) was added as an enabling organizational factor since studies indicate that use of technology influences the perception of work intensity [52,53]. Digital work may also facilitate health if it optimizes work organization [54]. In contrast, work intensity was chosen as an impeding organizational factor for HPM participation.

Work intensity was included as a continuous variable, while all remaining included variables were dichotomous. Further variables were not included to avoid overloading the final model. Missing values were not imputed. The odds ratio (OR), corresponding 95% confidence intervals (95% CI), *p*-values, Cox–Snell pseudo-R^2^, and Nagelkerke’s pseudo-R^2^ were estimated for all models. The statistical analysis was conducted using IBM SPSS Statistics version 27 for Windows (IBM, Armonk, NY, USA).

#### 2.5.2. Qualitative Analysis

For qualitative analysis of open-ended answers, directed and conventional content analysis was used [55]. Content analysis allows flexible analysis of text data and a subsequent quantitative perspective on findings. First, we applied a deductive approach: We used the open-ended questions in the survey as the basis for coding and structuring analysis by deducing the main category names from these questions. More specifically, responses to the following questions were analyzed: “From your perspective, which improvements are necessary to make your working conditions more health-promoting?“ (1), “What keeps you from doing so [participating in HPMs] currently?” (2), and “Which HMPs would interest you?” (3). Names and definitions for subcategories resulting from this analysis were not predetermined, but emerged from the data. One of the authors (K.S.) and a graduate student in rehabilitation sciences conducted qualitative analysis. K.S. reviewed coding and content classification. The coding scheme is available in Supplementary File Table S1. Frequencies of coded texts were converted into descriptive percentages for better interpretation [45]. Finally, example responses were chosen for each subcategory and translated into English. Qualitative data were organized using MAXQDA 2018 (VERBI Software, Berlin, Germany).

## 3. Results

### 3.1. Sociodemographic, Health-, and Work-Related Characteristics

A total of 179 managers served as the analysis sample. Due to occasional missing responses, the sample size varied depending on the available data for certain variables (between 164 and 179 full responses). Table 1 presents the sociodemographic, health-, and work-related characteristics of the sample. The majority of managers (84.3%) was 45–64 years old and male (67.1%). Respondents mostly worked in middle level management (65.2%) and had an average managerial experience of 11.94 years (SD: 6.67). Regarding health-related characteristics, the mean subjective wellbeing of managers was 59.98 on a range of 0 to 100 (SD: 20.99). Out of these, 32.2% of managers were classified with low wellbeing (WHO-5-score ≤ 50) and 67.8% with high wellbeing (WHO-5-score > 50). On average, managers considered their current health status fair (Mean: 3.56; SD: 0.86, on a scale of 1 to 5). The average work intensity of managers was perceived as higher (Mean: 3.15; SD: 0.4, on a scale of 1 to 4).

### 3.2. Quantitative Descriptive Findings: Working Conditions and HPM Participation

**Figure 1:**
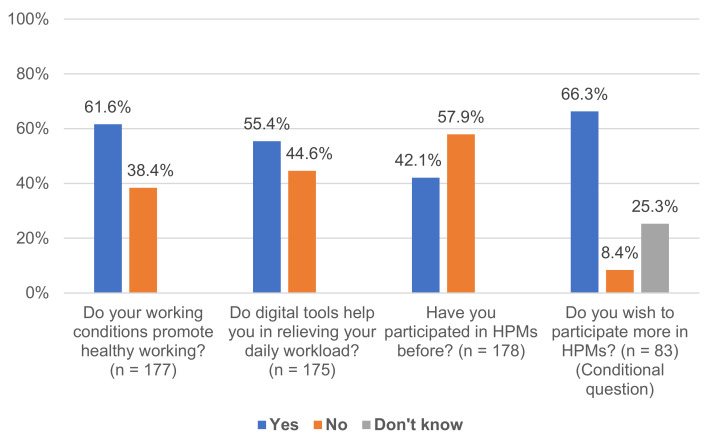
Frequencies of Managers’ Perceived Working Conditions and HPM Participation.

**Figure 2:**
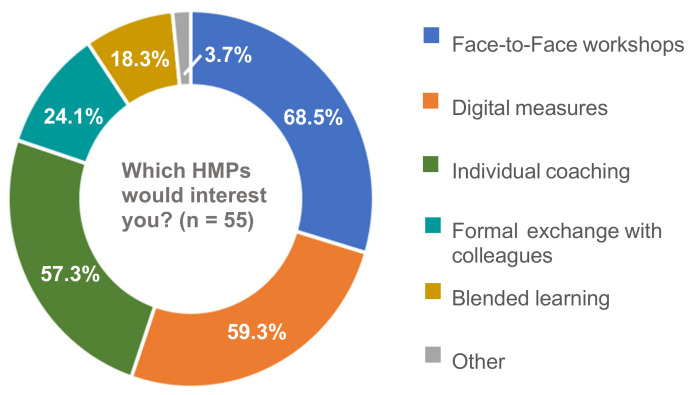
Frequencies of Managers’ Interest in HPM Modes (conditional question, multiple response option).

More than half of managers (61.6%, *n* = 109) considered their working conditions health-promoting and thought digital tools helped relieve their daily workload (55.4%, *n* = 97, see Figure 1). Similarly, the majority of managers (57.9%, *n* = 103) had not participated in HPMs before. Managers, who were asked if they wished to participate in HPMs more often (*n* = 83), mostly affirmed that wish (66.3%, *n* = 55). The remaining managers did not know (25.3%) or declined (8.4%). Across all HPM modes, managers who wished to participate in HPMs more often preferred face-to-face workshops (68.5%), digital measures (59.3%), and individual coaching (57.3%) (multiple response option). Respondents were less interested in formal exchange with colleagues, blended learning, or other HPM modes (see Figure 2).

### 3.3. Logistic Regression: Association of Individual and Organizational Variables with HPM Participation

Table 2 presents the results of the stepwise logistic regression analyses for included variables associated with managers’ previous HPM participation. The overlap of complete values for all included variables resulted in an analysis sample of *n* = 160. Model 1 included age group, gender, and management level as predisposing individual characteristics. Model 2 added wellbeing (WHO-5) as an indicator for health services need. Neither model 1 nor model 2 were significant. Model 3 included “workload relief through digital tools” as an enabling resource and work intensity as an impeding factor for previous HPM participation. Model 3 was significant (Chi-Square = 13.43, *p* = 0.037). While work intensity did not show a statistically significant association, “workload relief through digital tools” showed a significant association with previous HPM participation (OR: 2.84, 95% CI: 1.42–5.66, *p* = 0.003). Hence, managers, who thought digital tools helped relieve their daily workload, had about 2.9 times higher odds of having participated in HPMs before. Regarding fit of the final model, values for Cox–Snell pseudo-R2 (0.081; Cohens f^2^: 0.088) and Nagelkerke’s pseudo-R^2^ (0.108; Cohens f^2^: 0.12) indicated a small effect [56,57].

### 3.4. Qualitative Findings: Working Conditions and HPM Participation

The qualitative analysis resulted in 410 coded text segments, three categories, and 15 subcategories. Table 3 presents categories, subcategories, frequencies of coded text segments, and response examples for open-ended questions.

Out of all participants, 113 managers (63.1%) answered the question “From your perspective, which improvements are necessary to make your working conditions more health-promoting?”. This yielded 194 coded texts in the first category. The most frequently reported suggestions concerned work facilities, commute, and HPM offers (37.1%), as well as high workload and available time (32%). Leadership, teamwork, and social support made up 17.5% of managers’ suggestions, while 10.3% concerned corporate culture. Only 3% of texts stated that improvements are not necessary regarding health-promoting working conditions.

Examples for the most frequently mentioned digital tools that help relieve managers’ daily work included specific software such as office tools, web conference tools, instant messaging clients, and cloud systems. Fifty-five managers wished to participate in HPMs more often. Out of these, 49 managers provided a written answer to the conditional question “What keeps you from doing so [participating in HPMs] currently?”.

Furthermore, out of 93 managers, who knew about the mindfulness training offered in the company, 40 managers provided written answers about reasons for nonparticipation or lacking interest in the training. These findings resulted in the category “Barriers to HPM participation”, yielding 97 coded texts. The most mentioned barriers were conflicting schedules and daily workload (26%), lack of time (25%), and lack of specific information and knowledge (25%). Lacking demand and other reasons (12.4% each) were stated less frequently. In responding to the conditional question “Which workplace HPMs have you attended?”, 70 managers (of 75 who had attended HPMs before) named one measure or more. This yielded 115 coded texts. Measures that were most frequently mentioned addressed resilience, mindfulness, and stress relief (31.3%). This was followed by measures addressing physical fitness and health at work (25.5%) and medical measures and occupational safety measures (20.9%). Measures for healthy leadership (12.2%) and other measures, such as coaching, were mentioned less frequently (10.5%).

## 4. Discussion

This study’s primary objective (1) was to examine managers’ self-reported participation in HPMs and factors associated with their HPM participation. The secondary objective (2) was to examine managers’ perceptions of their working conditions. In light of previous evidence, we will first summarize and discuss findings for objective (1) and then objective (2). Quantitative and qualitative findings will be discussed for each objective using an integrative approach by complementing findings that are consistent or conflicting. Our sample corresponds with representative gender distributions of managers in Germany [58]. However, the older age group is more predominant compared to larger managerial samples [59,60].

### 4.1. Managers’ HPM Participation (1)

Quantitative findings revealed that 57.9% (*n* = 103) of managers in the sample had not participated in HPMs yet. Most managers, who had not participated in HPMs before, wished to participate more often. Despite a large range in participation levels reported in previous research, managers’ HPM participation rate of 42% in this study lies slightly above the median employee participation rate of 33% that was identified in a systematic review of Robroek et al. (2009) [15]. While a systematic review does not exist for Germany, a recent representative study demonstrated that 20–30% of employees in Germany utilize workplace HPMs [16]. Thus, our findings concur with studies indicating that managers may be more likely to report and participate in available HPMs [17,18,19]. However, due to our restricted subsample of managers, a selection effect is likely. Interestingly, previous participation in mental health measures was mentioned most often by managers in qualitative findings. Indeed, a fairly large percentage of managers (32%) was classified with low wellbeing (WHO-5-score ≤ 50)—a mark that has been used in clinical studies to initiate a screening diagnosis of depression [48]. Prior studies using the WHO-5 revealed a similar yet smaller rate of 19–25% of managers having low wellbeing [61,62,63]. These findings could indicate a higher need for mental health measures among the occupational group of managers.

### 4.2. Association of Individual and Organizational Factors with Managers’ HPM Participation (1)

Using regression analysis, we found that managers, who thought digital tools help relieve their daily workload, were 2.9 times more likely to have participated in HPMs before. Sociodemographic variables, wellbeing, and work intensity did not show a significant association with previous HPM participation. Qualitative findings revealed that managers reported high workload, lack of time, lack of knowledge, and lack of demand as barriers for HPM participation.

For brevity, only findings from the final regression model will be discussed. Andersen’s model explains health service utilization by predisposing, enabling, and need factors at individual and contextual levels [31]. Most factors included in the regression analysis, i.e., age, gender and management level, fall into the predisposing factors on the individual level. Wellbeing was used as a need factor on the individual level. These variables were not significant in our models. The nonsignificant associations add to the conflicting evidence for whether sociodemographic variables [15,18,33,34], health status and need [15,16,31,32,35,37] influence HPM participation. Furthermore, “workload relief through digital tools” was assigned as an enabling resource and work intensity was assigned as an impeding factor on the organizational level in our final regression model. Since roughly 50% of analyzed factors of HPM participation do not reach statistical significance in studies [15], it is surprising that solely “workload relief through digital tools” displayed a significant association. This could be due to the small sample or presence of other confounders. Barriers to managers’ HPM participation mentioned in qualitative findings may hint at additional impeding factors. These could be assigned to the contextual, i.e., organizational level (“workload”, “lack of time”, and “lack of knowledge”), and a lacking need on managers’ individual level (“no perceived demand”). The qualitatively reported barriers align with prior evidence indicating that time restrictions [24,37], knowledge about HPM availability [25,36,37], and fit to needs and preferences [34] influence HPM participation. Findings of the most frequent category “Suggested improvements for more health-promoting working conditions” do not address HPM participation. Following the logic of Andersen’s model however, one could argue whether its subcategories (work facility traits, high workload, social support, and corporate culture) can be allocated to enabling resources or impeding factors for HPM participation on the organizational level. One particularly interesting question for future studies is the role of superiors’ social support for managers’ HPM participation, as managers hold a special position between their own superiors and employees. This requires further exploration and testing.

### 4.3. Managers’ Working Conditions (2)

Quantitative findings revealed that 61.6% of managers (*n* = 109) considered their work health-promoting. The average work intensity of managers in our sample was high (3.15 on a four-point scale). In qualitative findings, managers reported that work facility traits, high workload, social support, and corporate culture should be improved to make their working conditions more health-promoting. 

Based on the quantitative findings, one could argue most managers in the sample may perceive their work as health-promoting while having high work demands [6]. Future studies could investigate whether managers generally consider their work more health-promoting compared to other occupational groups. Still, the qualitative findings provide indications for possible improvements of managers’ working conditions in the ICT sector, such as work facility traits, working structure, and the social and cultural environment. One particularly interesting finding is managers’ ICT exposure. Criticism of workload and information overload was prominent in the subcategory “workload and time”. Additionally, 45% of managers denied that digital tools help relieve their daily workload. These findings may strengthen evidence that managers have higher odds of exposure to ICT demands [8] and choice overload [62]. The association of workload and use of digital work media might be a relevant dimension for improving healthy working conditions for managers in particular. 

## 5. Strengths and Limitations

There are various limitations to this study that need to be acknowledged when interpreting the findings. This study used a cross-sectional design and thus cannot represent longitudinal causal relationships of variables. Explanatory power and validity of the noncomplex statistical regression are restricted to self-reported variables and the restricted subsample. Data saturation is limited due to the sample size and use of conditional questions in the survey. Reminders (e.g., according to Dillman [64]) may have enhanced the response rate (10.5%), though participation in workplace surveys is generally moderate or low [65]. Furthermore, managers’ HPM participation was addressed within their general perceived past and selection bias likely created an overestimation of the participation rate. For future studies, using probability sampling is recommended to decrease bias and increase the representativeness of samples. We face the risk of confounding, as further possibly relevant variables were not included in regression analyses. Nonetheless, this study adds value by contributing to the scarcely investigated HPM participation of managers. One particular strength of the study is the mixed-methods survey approach, consolidating the comprehensiveness of findings by supplementing qualitative and quantitative findings. The dependence on secondary data and the strong impact of the population and the study context on variable associations should be taken into account [66]. As this study was quantitatively driven, future mixed-methods studies should emphasize qualitative data gathering and qualitative analysis informed by theoretical constructs of HPM participation to enhance the logical reasoning of findings. While the study was conducted in just one ICT company and the generalizability of the findings may be limited, the investigated company is fairly large and managers were located at a variety of departments across Germany. Future studies are advised using larger samples including various organizations, more validated measures, and longitudinal designs for these purposes.

## 6. Conclusions

Our mixed-methods study provides insights into managers’ participation in workplace health promotion measures and perceptions of their working conditions. Managers, who thought digital tools help relieve their daily workload, were more likely to have participated in HPMs. Workload, time, lack of knowledge, and demand were reported as participation barriers. Furthermore, managers reported that work facility traits, workload, social support, and corporate culture should be improved to make their working conditions more health-promoting. Though future studies need to confirm these findings, this study provides starting points to improve managers’ work environment and participation in health promotion measures. Given their impact as role models, it is important to assess whether occupational health management and health promotion measures reach managers adequately. In light of digitalization and remote work, researchers and corporate health professionals are prompted to pay closer attention to managers’ working conditions to suit this particular target group in future health promotion measures.

## Figures and Tables

**Table 1:** Descriptive Statistics of Managers’ Sociodemographic, Health-, and Work-Related Characteristics.

Variable	*n*	Item	Distribution			%
**Sociodemographic Characteristics**						
Age Group (yrs)	178					100
		25–44		26		14.6
		45–64		150		84.3
		n. s.		2		1.1
Gender	173					100
		Female		57		32.9
		Male		116		67.1
Management Level	178					100
		Top		39		21.9
		Middle		116		65.2
		Low		19		10.7
		n. s.		4		2.2
	*n* (%)	Mean	SD	Minimum	Maximum	Median
Managerial Experience (yrs)	178 (100)	11.94	6.67	1	35	11.00
**Health- and Work-Related Characteristics**						
	*n* (%)	Mean	SD	Minimum	Maximum	Median
Wellbeing	174 (100)	59.98	20.99	0	100	64.0
Low	56 (32.2)					
High	118 (67.8)					
Health Status	175 (100)	3.56	0.86	1	5	4
Work Intensity	179 (100)	3.15	0.4	2	4	3.14

Abbreviations: yrs = Years; n. s. = Not Specified; SD = Standard Deviation. Note: Age groups “younger than 18 years”, “18–24 years”, and “65 years and older” were not selected by participants and are thus not presented.

**Table 2:** Progressively Adjusted Logistic Regression Models for Variables Associated with Managers’ Previous HPM Participation (*n* = 160).

	Model 1 (Crude Model)			Model 2			Model 3(Full Model)		
Variables (*n* = 160)	OR	95% CI	*p*	OR	95% CI	*p*	OR	95% CI	*p*
Age (refcat: 45–64 yrs)	0.527	[0.206, 1.346]	0.181	0.528	[0.206, 1.348]	0.181	0.437	[0.166, 1.153]	0.094
Gender (refcat: male)	1.700	[0.859, 3.366]	0.128	1.718	[0.866, 3.411]	0.122	1.528	[0.752, 3.104]	0.241
Management level (refcat: low)	0.828	[0.378, 1.811]	0.636	0.820	[0.374, 1.797]	0.621	0.742	[0.330, 1.668]	0.471
Wellbeing (refcat: low)				1.163	[0.584, 2.313]	0.668	0.905	[0.427, 1.918]	0.794
Workload relief through digital tools (refcat: no)							2.838	[1.422, 5.661]	0.003 *
Work intensity							0.843	[0.353, 2.013]	0.700
Goodness of fit	**Model 1**			**Model 2**			**Model 3**		
Cox–Snell pseudo-R^2^	0.025			0.026			0.081		
Nagelkerke’s pseudo-R^2^	0.034			0.035			0.108		

Note: refcat = Reference Category; yrs = Years; OR = Odds Ratio; CI = Confidence Interval; * *p* < 0.05.

**Table 3:** Categories, Frequencies, and Response Examples: Managers’ Suggestions for Health-Promoting Working Conditions, Barriers to HPM Participation, and Previous HPM Participation.

Category	% (*n*)	Example Responses
**Suggested improvements for more health-promoting working conditions**	100 (194)	
Work facilities, commute, and HPM offers	37.1 (72)	Fixed workplaces; better (open-plan) office design—more quiet zones, better indoor climate, more foliage plants; less traveling; more sports and exercise offers (during working hours); significant improvement of culinary selection for healthier nutrition; water dispensers instead of coffee machines
Workload and time	32.0 (62)	High workload, too many simultaneous topics; less stress and pressure; reduction in complexity and ambiguity; clearer prioritization; conflicting schedules; less work compression on each individual; less but better prepared information; breaks and rest periods are still often considered a weakness…; better work–life balance for managers, too; respect private times; no calls and mails after 6 p.m.
Leadership, teamwork, and social support	17.5 (34)	Rules for teamwork; more appreciation; more respect towards staff; positive, motivating, inspiring atmosphere—even in hard times; more face-to-face meetings and less virtual teamwork; interaction with colleagues; selfishness of individuals should be fought instead of encouraged; change in management style of some colleagues
Corporate culture	10.3 (20)	The company should develop a culture in which employee health is a real value; the human being must be emphasized in the company again; improve feedback culture; rejuvenation of the organization; more digitalization in the ENTIRE company, not only in parts; we do a lot regarding overtime compensation for employees, for managers there are no comparable compensations. In my opinion, it always comes across as somewhat strange when managers say to their manager, “But I have worked quite a lot of overtime now…”, that is not our corporate culture
Working conditions are ideal	3.1 (6)	Nothing. It is already ideal; I think the shortage rather lies within me, less within the employer/working conditions; There are few things to improve regarding working conditions. I should improve my mindfulness, awareness, and health orientation (taking breaks, eating, managing my energy)…
**Barriers to HPM participation ^CQ^**	100 (97)	
Conflicting schedules and daily workload	25.8 (25)	Too many other things to do; scheduling conflicts; constant need to prioritize daily business and special tasks; high workload
Lack of time	24.7 (24)	TIME!; time shortage; time resources not available
Lack of specific information and knowledge	24.7 (24)	There is a lack of targeted addressing and targeted appointment offers; Appointments + offers not known, no communication about offers across locations; I did not know this existed
No perceived demand	12.4 (12)	Need not recognized
Other	12.4 (12)	I wonder, whether the real working conditions in the company allow an implementation of what has been learned; no offer at the location or in working vicinity
**Previous HPM participation ^CQ^**	100 (115)	
Resilience, mindfulness, and stress relief	31.3 (36)	Resilience workshop; mindfulness workshop; work–life balance seminar; stress management seminar
Physical fitness and health at work	25.5 (29)	Mobile fitness coach; back training; company fitness center; company health days
Medical measures and occupational safety	20.9 (24)	Medical checkup at the workplace; vaccinations; ergonomics and safety training
Leadership	12.2 (14)	Healthy leadership; virtual leadership; leading in agile environments
Other	10.5 (12)	Personal coaching; coaching for business unit; online training

Note: % (*n*) refers to the number of coded texts. Frequencies of coded texts do not equal frequencies of quantitative cases; CQ = The category is based on a conditional question.

## Data Availability

The datasets generated and/or analyzed during the current study are not publicly available as they contain sensitive data that belong to the ICT company. Quantitative data are available in anonymized form on reasonable request.

## Supplementary Materials

The following are available online at https://www.mdpi.com/article/10.3390/ijerph18189708/s1, Table S1: Coding Scheme: Suggestions for Health-Promoting Working Conditions, Barriers to HPM Participation, and Previous HPM Participation.

## Abbreviations

HPMs	Health Promotion Measures
ICT	Information and Communication Technologies
