# Supplementary material for: How Managers Perceive and (Do Not) Participate in Health Promotion Measures—Results from a Cross-Sectional Mixed-Methods Survey in a Large ICT Company
Source: Int J Environ Res Public Health. 2021 Sep 15;18(18):9708. doi: 10.3390/ijerph18189708 (PMC8468359; doi:10.3390/ijerph18189708)
Supplement: Supplementary file 1 [file ijerph-18-09708-s001.zip › ijerph-1360729-supplementary.pdf]

Table S1. Coding Scheme: Suggestions for Health-Promoting Working Conditions, Barriers to HPM Participation and Previous HPM Participation.

Category	Definition
**Suggested improvements for more health-promoting working conditions**	This category includes all statements that express suggestions, wishes, ideas or criticism regarding working conditions. This also category includes statements describing if and how working conditions are health-promoting or not.
**Subcategory**	**Definition**
Work facilities, commute, and HPM offers	This subcategory includes all suggestions, wishes, ideas or criticism regarding the location, commute, and physical working environment. This includes statements about room layout, equipment, workplace organization (e.g. fixed vs. flexible workplace), noise level, ergonomics, work-related travel or commuting. This subcategory also includes suggestions or wishes for additional workplace health promotion measure offers.
Work load and time	This subcategory includes all statements that indicate a high workload, information overload, high pressure, high demands, versatility and complexity of tasks. This subcategory also includes statements about managers' work-life-balance (e.g. breaks, vacation), its implementation in the company, and managers' accessibility outside working hours. Suggestions, wishes, and ideas for relief of workload and demands are included as well.
Leadership, teamwork, and social support	This subcategory includes all statements on interpersonal relationships, quality of interaction, behavior and competencies of colleagues and superiors, quality of leadership and teamwork, and social support at the workplace. This subcategory also includes statements about forms of teamwork (e.g. virtual vs. on-site collaboration).
Corporate culture	This subcategory includes all statements that concern the corporate culture, values, norms, structures and processes of the company as a whole (e.g. change processes, restructuring, digitalization).
Working conditions are ideal	This subcategory includes all statements expressing that the working conditions or their impact on health are non-problematic, positive, or ideal. This subcategory also includes statements emphasizing the individual's responsibility for health-promoting behavior.
**Barriers to HPM participation**	This category includes all statements on work-related or individual factors that impede participation in workplace health promotion measures.
**Subcategory**	**Definition**
Conflicting schedules and daily workload	This subcategory includes all statements indicating that the priority of work tasks, deadlines, high work pressure or the complexity of daily work routines impede participation in workplace health promotion measures.
Lack of time	This subcategory includes all statements indicating that a lack of time impedes participation in workplace health promotion measures.
Lack of specific information and knowledge	This subcategory includes all statements indicating that the amount, transfer or lack of information, and resulting unawareness about health promotion measures impede participation therein (e.g. lack of information, unmanageable digital information overload).
No perceived demand	This subcategory includes all statements indicating there is no perceived demand for participation in workplace health promotion measures or certain measures have already been attended in the past.
Other	This subcategory includes all statements about barriers to participation in workplace health promotion measures that cannot clearly be assigned to other subcategories. Examples include a perceived lack of possibilities for implementing learning outcomes in everyday work or inadequate measure offers.

**Previous HPM participation**	This category includes all statements referring to workplace health promotion measures that managers attended in the past. Names of measures and description of measure contents are included.
**Subcategory**	**Definition**
Resilience, mindfulness, and stress relief	This subcategory includes all statements referring to workplace health promotion measures aiming at mental health (e.g. stress management, mindfulness, resilience or work-life-balance).
Physical fitness and health at work	This subcategory includes all statements referring to workplace health promotion measures aiming at physical fitness and general health at work (e.g. company health days).
Medical measures and occupational safety	This subcategory includes all statements referring to use of medical services at the workplace and measures aiming at occupational safety (e.g. vaccinations, medical checkups).
Leadership	This subcategory includes all statements referring to workplace health promotion measures aiming at leadership quality and healthy leadership.
Other	This subcategory includes all statements about previously attended workplace health promotion measures that cannot clearly be assigned to the other subcategories (e.g. coaching, online training).

Note: HPM = Workplace Health Promotion Measure
